# Protease-associated cellular networks in malaria parasite *Plasmodium falciparum*

**DOI:** 10.1186/1471-2164-12-S5-S9

**Published:** 2011-12-23

**Authors:** Timothy G Lilburn, Hong Cai, Zhan Zhou, Yufeng Wang

**Affiliations:** 1 Department of Bacteriology, American Type Culture Collection, Manassas, VA 20110, USA; 2 Department of Biology, University of Texas at San Antonio, San Antonio, TX 78249, USA; 3 College of Life Sciences, Zhejiang University, Hangzhou 310058, PR China; 4 South Texas Center for Emerging Infectious Diseases, University of Texas at San Antonio, San Antonio, TX 78249, USA

## Abstract

**Background:**

Malaria continues to be one of the most severe global infectious diseases, responsible for 1-2 million deaths yearly. The rapid evolution and spread of drug resistance in parasites has led to an urgent need for the development of novel antimalarial targets. Proteases are a group of enzymes that play essential roles in parasite growth and invasion. The possibility of designing specific inhibitors for proteases makes them promising drug targets. Previously, combining a comparative genomics approach and a machine learning approach, we identified the complement of proteases (degradome) in the malaria parasite *Plasmodium falciparum *and its sibling species [1-3], providing a catalog of targets for functional characterization and rational inhibitor design. Network analysis represents another route to revealing the role of proteins in the biology of parasites and we use this approach here to expand our understanding of the systems involving the proteases of *P. falciparum*.

**Results:**

We investigated the roles of proteases in the parasite life cycle by constructing a network using protein-protein association data from the STRING database [4], and analyzing these data, in conjunction with the data from protein-protein interaction assays using the yeast 2-hybrid (Y2H) system [5], blood stage microarray experiments [6-8], proteomics [9-12], literature text mining, and sequence homology analysis. Seventy-seven (77) out of 124 predicted proteases were associated with at least one other protein, constituting 2,431 protein-protein interactions (PPIs). These proteases appear to play diverse roles in metabolism, cell cycle regulation, invasion and infection. Their degrees of connectivity (i.e., connections to other proteins), range from one to 143. The largest protease-associated sub-network is the ubiquitin-proteasome system which is crucial for protein recycling and stress response. Proteases are also implicated in heat shock response, signal peptide processing, cell cycle progression, transcriptional regulation, and signal transduction networks.

**Conclusions:**

Our network analysis of proteases from *P. falciparum *uses a so-called guilt-by-association approach to extract sets of proteins from the proteome that are candidates for further study. Novel protease targets and previously unrecognized members of the protease-associated sub-systems provide new insights into the mechanisms underlying parasitism, pathogenesis and virulence.

## Background

Malaria remains a major threat to health and economic development in endemic countries, infecting 300-500 million people yearly and claiming 1-2 million deaths, primarily of young children. Symptoms of malaria include high fever, shaking chills, headache, vomiting, and anemia. If left untreated, malaria can quickly become life threatening by disrupting the blood supply to vital organs. Malaria is caused by a group of parasites from the genus *Plasmodium*. Five species, *P. falciparum*, *P. vivax*, *P. malariae*, *P. ovale*, and *P. knowlesi*, are known to cause the disease in humans. *P. falciparum *is the most devastating and widespread species.

No effective anti-malaria vaccines are available for use in humans [13]. For decades, the management of malaria has relied heavily on chemotherapy, which uses a limited number of drugs. However, the rapid evolution and spread of drug resistance in parasites has led to an increase in morbidity and mortality rates in malaria endemic regions. The development of new drug/vaccine targets is urgently needed.

Thanks to the completion of the genome sequencing projects for *P. falciprum *and its sibling species [14-19], a novel array of proteins have been proposed as potential drug targets, including (1) proteins like 1-deoxy-D-xylulose 5-phosphate (DOXP) reductoisomerase [20,21], and apicoplast gyrase [22] that are located in the apicoplast, an organelle with its origin close to the chloroplast; (2) kinases such as cyclin-dependent protein kinases (Pfmrk) [23] and the plant-like calcium-dependent protein kinase (PfCDPK5) [24]; (3) transporters involved in drug resistance and nutrient acquisition from the host [25-30], and (4) proteases.

Proteases are a group of enzymes that degrade proteins by breaking peptide bonds. They are attractive antimalarial targets due to their indispensible roles in parasite development and invasion [31,32]. Previously we predicted the protease complement (degradome) in the malaria parasite *P. falciparum *and its four sibling species using a comparative genomics approach and a support vector machine (SVM)-based, supervised machine learning approach [1-3]. This catalog revealed a new line of novel proteases for functional characterization. Studies on malarial proteases have been focused on biochemical and molecular characterization [33-46], structural modeling and analysis [47,48], and inhibitor design and screening [49-59]. Although significant progress has been made, much remains to be learned about the roles played by these proteins, including how they interact with other proteins in space and time to coordinate important aspects of growth, transmission, invasion, response to drug treatment and pathogenesis of this devastating pathogen.

One approach to gaining wider views on the roles of proteins in biological systems relies on network biology. Known and inferred protein associations are used to build a network of proteins, thus establishing a map of all the associations in the organism and allowing deductions to be made as to the role of proteins that are poorly understood and poorly annotated. Clearly, both proposed and demonstrated protein-protein associations could aid us in understanding the role of a protease in the parasite. Therefore, we constructed a network of *P. falciparum *proteins using the protein-protein association data from STRING database [4], and analyzed these data, in conjunction with the data from protein-protein interaction assays using the yeast 2-hybrid (Y2H) system [5], blood stage microarray experiments [6-8], proteomics [9-12], literature text mining, and sequence homology analysis. The topology of the protein-protein association network was analyzed and the results examined for information as to how the proteases may function within the parasite. Sets of proteins associated with specific proteases or protease families were extracted from the whole-cell network to create protease-associated subnetworks and five of these subnetworks were examined in detail. Novel protease targets and previously unrecognized members of some sub-systems could be postulated; these insights help us to better understand the mechanisms underlying parasite metabolism, cell cycle regulation, invasion and infection.

## Results and discussion

### Proteases are involved in complex networks

We downloaded and mined the protein-protein association data from the STRING database [4] involving proteins from *P. falciparum*. Seventy-seven (77) out of 124 predicted proteases were found in this set and were associated with at least one other protein, constituting 2,431 associations (Additional Files 1 and 2). Each association between a pair of proteins has a confidence score (S) ranging from 0.15 to 0.999 that was inferred from the evidence used to establish the association: 221 associations (9.1%) have high confidence scores (S>0.7), 432 associations (17.8%) have medium confidence scores (0.4≤S≤0.7), and strikingly, 1,778 associations (73.1%) have relative low confidence scores (0.15≤S<0.4). The large proportion of low-scored associations arises from the paucity of annotation data. Before the genome of *P. falciparum *was sequenced, only about 20 proteins had been characterized; after genome sequencing this number increased by two orders of magnitude, but over 60% of the predicted gene products in the genome still had no functional assignment [18] and ten years of subsequent effort have reduced this number to roughly 45% [60]. Consequently, information such as KEGG pathway assignments, PDB protein structures and reactome data, which tend to improve association scores, is scarce for *P. falciparum*. Therefore, our subsequent analysis will not exclude the associations with low confidence scores as they may well represent associations that have not been previously recognized.

**Figure 1:**
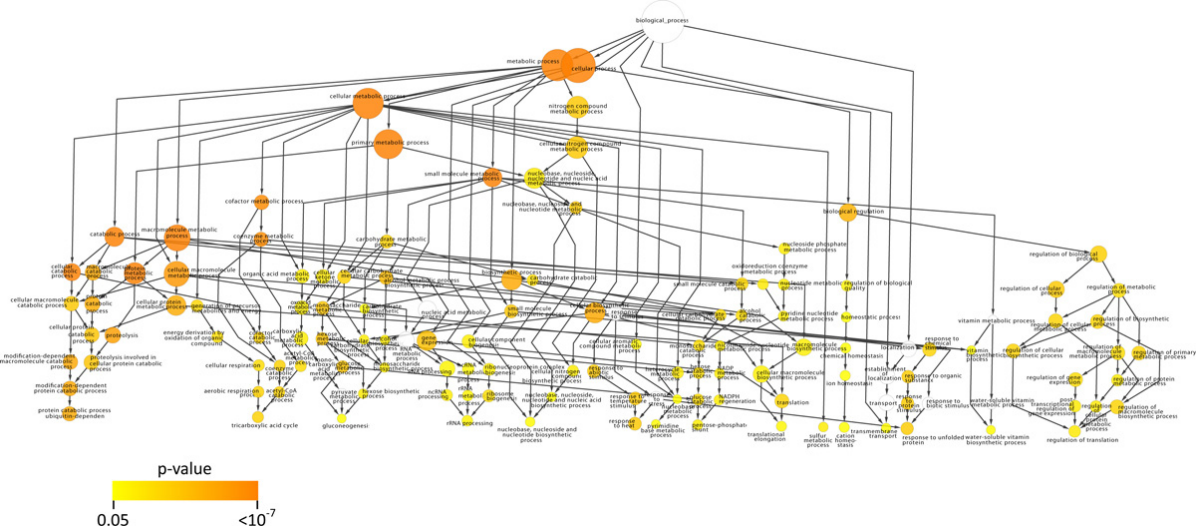
**A graphical representation of the results of a Gene Ontology analysis done using BiNGO**. The node size is proportional to the number of proteins represented by that GO term. The color represents the P-value for each enriched GO term as shown in the scale; white nodes are not enriched. The nodes are positioned to approximate their level in the Gene Ontology.

The degrees of connectivity vary among the 77 predicted proteases with protein-protein associations, ranging from one to 143 (Additional File 1). Twenty-four (24) putative proteases have less than five association partners, 13 have 5-10 partners, and 40 are highly connected with more than 11 partners, suggesting that proteases are involved in complex cellular networks. Functional enrichment analysis [61] revealed that 120 Gene Ontology (GO) terms were over-represented in these protease associations (p < 0.05) (Additional File 3). Figure 1 shows the distribution of functional categories in a hierarchical order: proteolysis (GO 6508) is, not surprisingly, enriched (p = 8.29*10^-6^), while the other most highly represented GO biological processes (p < 10^-5^) are related to cellular catabolic processes (GO 44248), protein metabolic processes (GO 19538), macromolecule metabolic processes (GO 43170), and cofactor and coenzyme metabolic processes (GO 51186 and 6732). This result reflects the involvement of proteases in fundamental biological processes, many of which have been established in the wet lab. Other processes that are moderately enriched (10^-5^<p < 0.05) included gene expression and response to endogenous and external stimuli such as heat, abiotic stimulus, organic substances, unfolded proteins, and protein stimuli. Five of the potentially most significant protease-associated sets of proteins are discussed in the following sections. They include the ubiquitin-proteasome system, the stress response system, the regulated intramembrane proteolysis system, the parasite egress network, and the signal peptidase network. These subnetworks were chosen because: (1) proteases are the central players in these networks; (2) These networks play crucial roles in parasite life cycle and are closely associated with adaptive phenotypes such as stress response, transcriptional regulation, pathogenesis, and virulence; (3) These networks are considered to be potential antimalarial targets as their disruption would cause deleterious effects on the growth or infectivity of the parasites.

### The ubiquitin-proteasome system (UPS)

The largest protease-associated network in *P. falciparum *is the ubiquitin-proteasome protein degradation system (UPS). The UPS is responsible for degrading unwanted or misfolded proteins and is believed to execute important roles in protein turnover and cell cycle regulation in a wide variety of organisms [62]. We previously identified a group of threonine proteases that form α- and β- subunits of the proteasome complex and two families of ubiquitin-specific hydrolases (C12 and C19) [1,63] (Additional File 1). The UPS pathway in *P. falciparum *has been deduced by Dr. Hagai Ginsburg (http://sites.huji.ac.il/malaria/maps/proteaUbiqpath.html), and involves two consecutive steps: (1) tagging the ubiquitin molecules to target proteins and (2) degradation of the tagged protein by the proteasome complex with release and recycling of ubiquitin. The major components of the UPS in *P. falciparum *are conserved with other eukaryotes. However, a growing body of evidence suggests that the UPS plays a critical role in the parasite-specific life style and it is therefore intriguing to unveil the proteins and pathways that are associated with or regulated by the UPS [64,65], as they may carry out functions specific to pathogenesis or virulence. We identified 1,148 associations in *P. falciparum *that involved 11 threonine proteases in the T1 family, two proteases in the C12 ubiquitin C-terminal hydrolase family, and six proteases in the C19 ubiquitin-specific protease family. One hundred and twenty-four (124) associations are protease-protease associations, and the remaining 1,024 associations involve non-protease partners. One hundred and sixty-four (164) of these associations have high confidence scores (S>0.7), the majority of which involve the association between catalytic components and regulatory components in the proteasome complex (Additional File 4).

**Figure 2:**
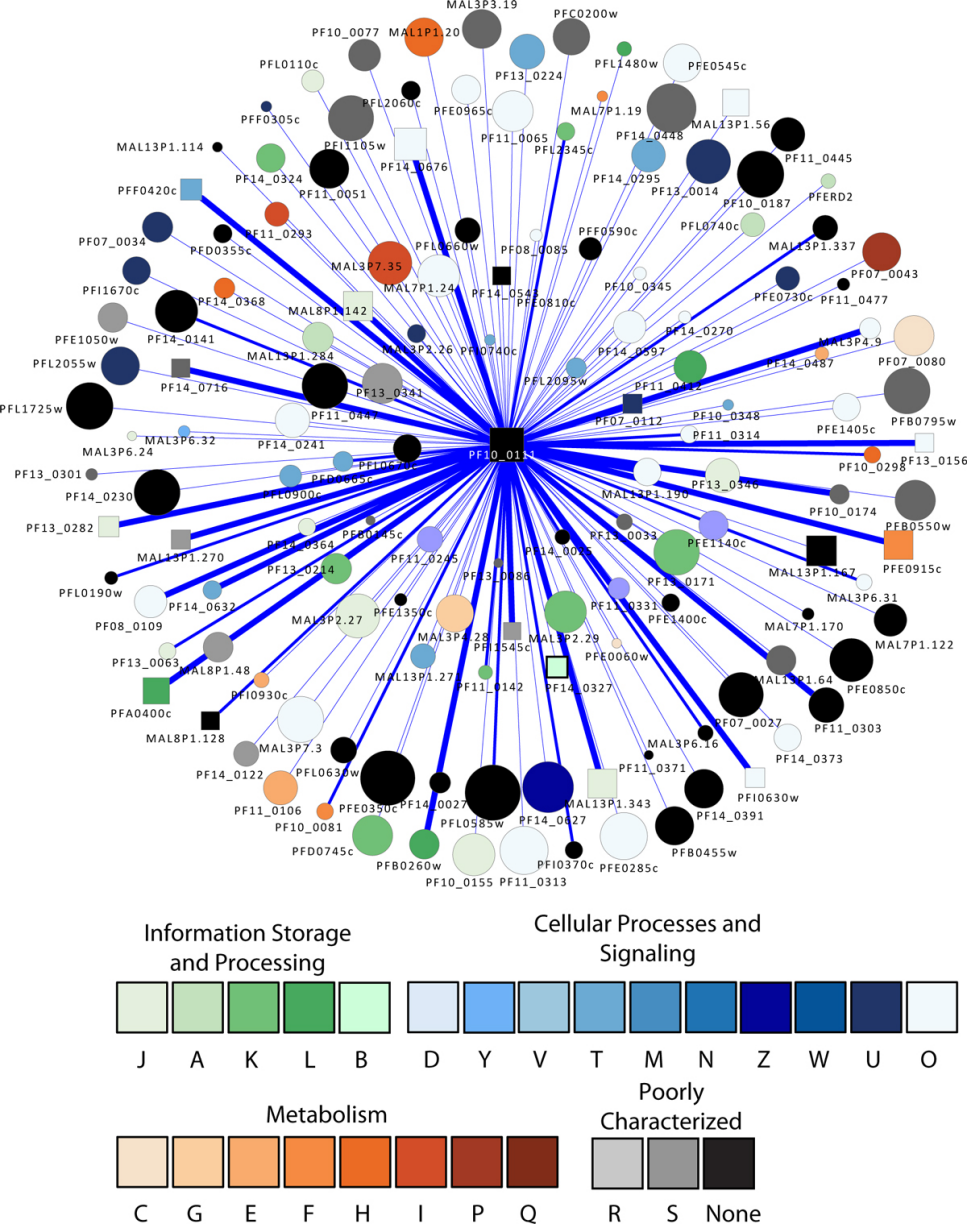
**A graph showing the proteins associated with PF10_0111**. This protease is the most highly connected member of the set of proteases found in the *P. falciparum *ubiquitin-proteasome protein degradation system. Square nodes represent proteases. Node size is proportional to the degree of the node. Nodes are colored according to their functional classification in the eggNOG database [122]. The COG categories are [123] (J) Translation, ribosomal structure and biogenesis, (A) RNA processing and modification, (K) Transcription, (L) Replication, recombination and repair, (B) Chromatin structure and dynamics, (D) Cell cycle control, cell division, chromosome partitioning, (Y) Nuclear structure, (V) Defense mechanisms, (T) Signal transduction mechanisms, (M) Cell wall/membrane/envelope biogenesis, (N) Cell motility, (Z) Cytoskeleton, (W) Extracellular structures, (U) Intracellular trafficking, secretion, and vesicular transport, (O) Posttranslational modification, protein turnover, chaperones, (C) Energy production and conversion, (G) Carbohydrate transport and metabolism, (E) Amino acid transport and metabolism, (F) Nucleotide transport and metabolism, (H) Coenzyme transport and metabolism, (I) Lipid transport and metabolism, (P) Inorganic ion transport and metabolism, (Q) Secondary metabolites biosynthesis, transport and catabolism, (R) General function prediction only, and (S) Function unknown. Confidence scores for the interactions among the nodes (S values from STRING) were divided into three groups - low (0.150-0.399), medium (0.400-0.700) and high (0.701-0.999); the groups are represented by thin, medium and heavy lines, respectively.

The protease with the highest connectivity is PF10_0111, a putative 20S proteasome beta subunit, which has 143 association partners (Figure 2). In addition to the proteasome components and ubiquitin conjugation enzymes, the other interacting proteins appear to be involved in a variety of activities (Table 1): (1) a nucleotide binding activity involving a Tat binding protein homolog (PFL2345c) which has an AAA ATPase domain; (2) cell cycle regulation involving MAL13P1.337, which is a putative protein in the Skp1 (S-phase kinase-associated protein 1) family. The Skp1 protein is an important component of the cyclin A-CDK2 S phase kinase complex in baker's yeast (*Saccharomyces cerevisiae*) [66] and directs cell cycle-regulated proteins to the kinetochore; (3) translation involving a number of ribosomal proteins such as 60S ribosomal proteins L40/UBI (PF13_0346) and L10 (PF14_0141), a putative translation initiation factor eIF-1A (PF11_0447), and a putative elongation factor 1 (EF-1) (PFC0870w); (4) transcriptional regulation involving a putative multiprotein bridging factor type 1 (MBF1) (PF11_0293). MBF1 is a transcriptional cofactor that bridges the TATA box-binding protein (TBP) and its specific regulatory proteins for transcriptional activation [67]; (5) membrane traffic regulation involving a putative rab specific GDP dissociation inhibitor (PFL2060c) [68].

**Table 1:** Representative *P. falciparum *proteins that are associated with PF10_0111, a putative 20S proteasome beta subunit with the highest connectivity. Protein-protein interactions revealed by yeast 2-hybrid assays are italicized.

Functional description	Protein accession number	Annotation
**Nucleotide binding**	PFL2345c	tat-binding protein homolog

**Cell cycle regulation**	MAL13P1.337	putative Skp1 family protein

**Transcriptional regulation**	PF11_0293	putative multiprotein bridging factor type 1

	*PF11_0477*	*CCAAT-box DNA binding protein subunit B*

**Translation**	PF13_0346	putative 60S ribosomal protein L40/UBI
	PF14_0141	putative 60S ribosomal protein L10
	PF11_0447	putative translation initiation factor eIF-1A
	PFC0870w	putative elongation factor 1 (EF-1)
	*PFE0350c*	*60S ribosomal protein L4*
	*PF14_0270*	*putative apicoplast ribosomal protein L15 precursor*
	*PF11_0245*	*putative translation elongation factor EF-1, subunit alpha*

**Protein transport**	PFL2060c	rab specific GDP dissociation inhibitor

**Protein modification**	*MAL7P1.19*	*putative ubiquitin transferase*

**Surface antigens**	*PF10_0345*	*merozoite surface protein 3*
	*PF10_0345*	*merozoite surface protein 3*
	*PF10_0348*	*duffy binding-like merozoite surface protein*

	*PFE0060w*	*parasite-infected erythrocyte surface protein*

**Unknown**	*MAL7P1.170*	*Plasmodium exported protein, unknown function*

Moreover, the yeast 2-hybrid assay using PF10_0111 as a bait revealed 15 PPI preys (Table 1), confirming that it is associated with (1) transcriptional regulation involving a CCAAT-box DNA binding protein subunit B (PF11_0477) containing a histone-like transcription factor domain, and (2) translation involving a putative translation elongation factor EF-1 subunit alpha (PF11_0245), a putative 60S ribosomal protein L4 (PFE0350c), and a putative ribosomal protein L15 precursor predicted to localize to the apicoplast (PF14_0270), a specific organelle of prokaryotic origin found in *Apicomplexa *parasites. PF10_0111 may also be associated with protein modifications involving a putative ubiquitin transferase (MAL7P1.19) [69] and chromatin fluidity involving a putative nucleosome assembly protein (PFI0930c).

Interestingly, PF10_0111 is shown to have PPI with three predicted surface antigens: (1) merozoite surface protein 3 (PF10_0345), which was shown by global RNA decay and nuclear run-on assays to serve a role in transcriptional regulation and RNA stabilization [70,71]; (2) a merozoite surface protein (PF10_0348). Domain analysis revealed a N-terminus Duffy binding domain that is present in the Duffy receptors expressing blood group surface determinants and a C-terminus SPAM (secreted polymorphic antigen associated with merozoites) domain, both of which have been implicated in parasite immune evasion, cytoadherence and pathogenesis [72,73]; (3) a parasite-infected erythrocyte surface protein (PFE0060w). The microarray and proteomics assays show that these three surface proteins are expressed at the invasive merozoite stage [6,8,10,11].

These results reflect much that is known about the UPS, but also suggest that it may also be associated with a variety of processes ranging from transcriptional regulation, translation, cell cycle progression, invasion, protein trafficking, and immune evasion. Not surprisingly, the UPS has become a promising antimalarial target. Various independent studies have shown that inhibition of proteasome activity can arrest parasite growth, and yet show limited toxicity to human cell lines [64,74,75].

### Stress response network

**Figure 3:**
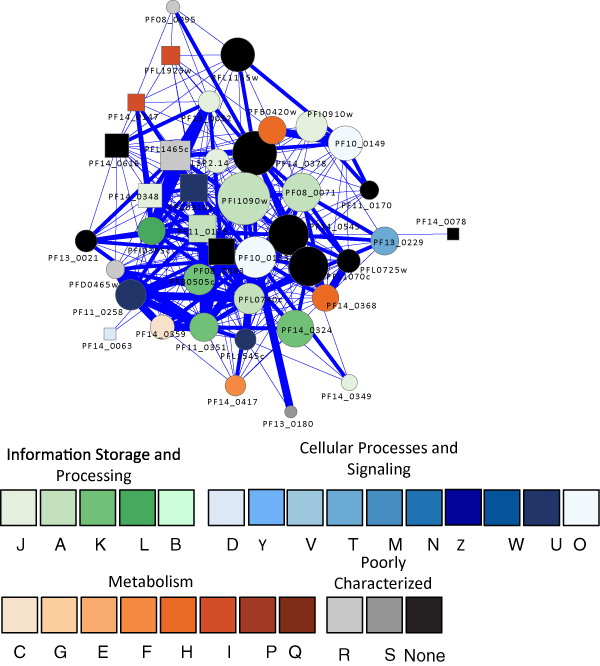
**The graph shows a subnetwork of proteins linked to stress responses in *P. falciparum***. It was detected using the MINE plug in for Cytoscape, which uses an agglomerative approach to search the topology of large networks for significant clusters. The visualization is as for Figure 2.

The adaptation of the malaria parasite to the host environment requires a rapid and effective response to diverse physiological signals and stress conditions, such as changes in temperature within hosts, nutritional challenges, host immune responses, antimalarial administration, and so on. One such adaptive network in the malaria parasite is the robust heat shock response system. During its life cycle, the parasite is transmitted from the mosquito vector (~25°C) to the human host (37°C), resulting in heat shock. Periodic fever, the patient's response to infection, also presents recurrent heat shock to the parasite. A comprehensive chaperone system has been identified in *P. falciparum *genome, accounting for 2% of the open reading frames (ORFs) [76]. The system is comprised of various chaperone proteins [77] and includes proteases that degrade misfolded proteins. We identified 344 associations involving five putative proteases in the ClpP endopeptidase family (S14), a lon protease PF14_0147 (S16), and an hslV protease PFL1465c (T1B). As shown in Figure 3, these proteases are associated with a large number of heat shock proteins (HSPs) including Hsp90, Hsp70, Hsp40, and DnaJ proteins. The protease having the highest degree of connectivity (80) in the heat shock response network is PFL1465c, a threonine protease hslV. In addition to the classical HSPs, it is associated with a wide variety of enzymes such as ubiE/COQ5 methyltransferase, rRNA methyltransferase, multiple tRNA synthetases, various phosphate isomerases, amino transferase, aldolase, and a number of kinases, suggesting it may have an important role in parasite metabolism. It is also associated with three other heat-shock response proteases (PFC0310c and PF08_0063 in the S14 ClpP endopeptidase family, and PF14_0147 in the S16 ATP-dependent protease lon family), an organelle processing peptidase in the M16 pitrilysin family (PFI1625c), a leucyl aminopeptidase in the M17 family (PF14_0439), and aminopeptidase P in the M24 family (PF14_0517); together, they form a complex protease degradation network. It is also interesting to note that this putative hslV protease appears to be linked to a second very important stress system in the malaria parasite that acts against oxidative challenges: Protease hslV is associated with the thioredoxin 1 protein (PF14_0545), a member of the thioredoxin system which controls cell redox homeostasis, and a putative Fe-superoxide dismutase (Fe-SOD, PF08_0071) which is critical for antioxidant defense. Because the malaria parasite is sensitive to oxidative stress, both the thioredoxin system and SOD have been considered as potential antimalaria targets [78]. Finally, our phylogenetic analysis revealed that this hslV protease (PFL1465) is of prokaryotic origin and there is no homolog in the human host, a desirable feature for drug targets [79-81]. A second heat shock response protease PfClpP (PFC0310c) was recently characterized [82,83]; protease inhibition assays have shown that it, along with other ATP-dependent chaperones, plays a crucial role in parasite growth and development. Furthermore, PfClpP is localized to the apicoplast, which is of cyanobacterial origin, making this protein an apicoplast-targeting antimalarial candidate. This protein is indeed highly connected with 69 association partners, including Hsp70, Hsp60, Hsp40, co-chaperones, and proteins involved in proteasome acitivities, replication, translation, protein biosynthesis, metabolism, and heat shock response, implying that its inactivation would have devastating consequences for the parasite.

### Signal transduction via the regulated intramembrane proteolysis (RIP) network

The common belief that proteases cleave peptide bonds in a water environment was challenged by the discovery of a set of proteases that conduct hydrolysis in the hydrophobic environment of cellular membranes [84]. During RIP, intramembrane proteases cleave transmembrane-spanning helical (TMH) segments of the substrates and release soluble effectors, many of which are signaling molecules, thereby triggering cascades of signal transduction pathways [85,86]. RIP is now believed to be a ubiquitous signaling mechanism in a wide variety of organisms from bacteria to humans [87]. The roles of RIP in the parasite life cycle have begun to be unraveled. Three families of membrane-tethered proteases involved in RIP have been identified in *P. falciparum*, including an aspartic signal peptide peptidase (PfAPP, PF14_0543) in the A22 presenilin family, eight rhomboid serine proteases (PfROMs) in the S54 family, and two putative Site-2 metallo proteases (S2Ps, PF13_0028 and PF10_0317) in the M50 family[1,88-93].

**Figure 4:**
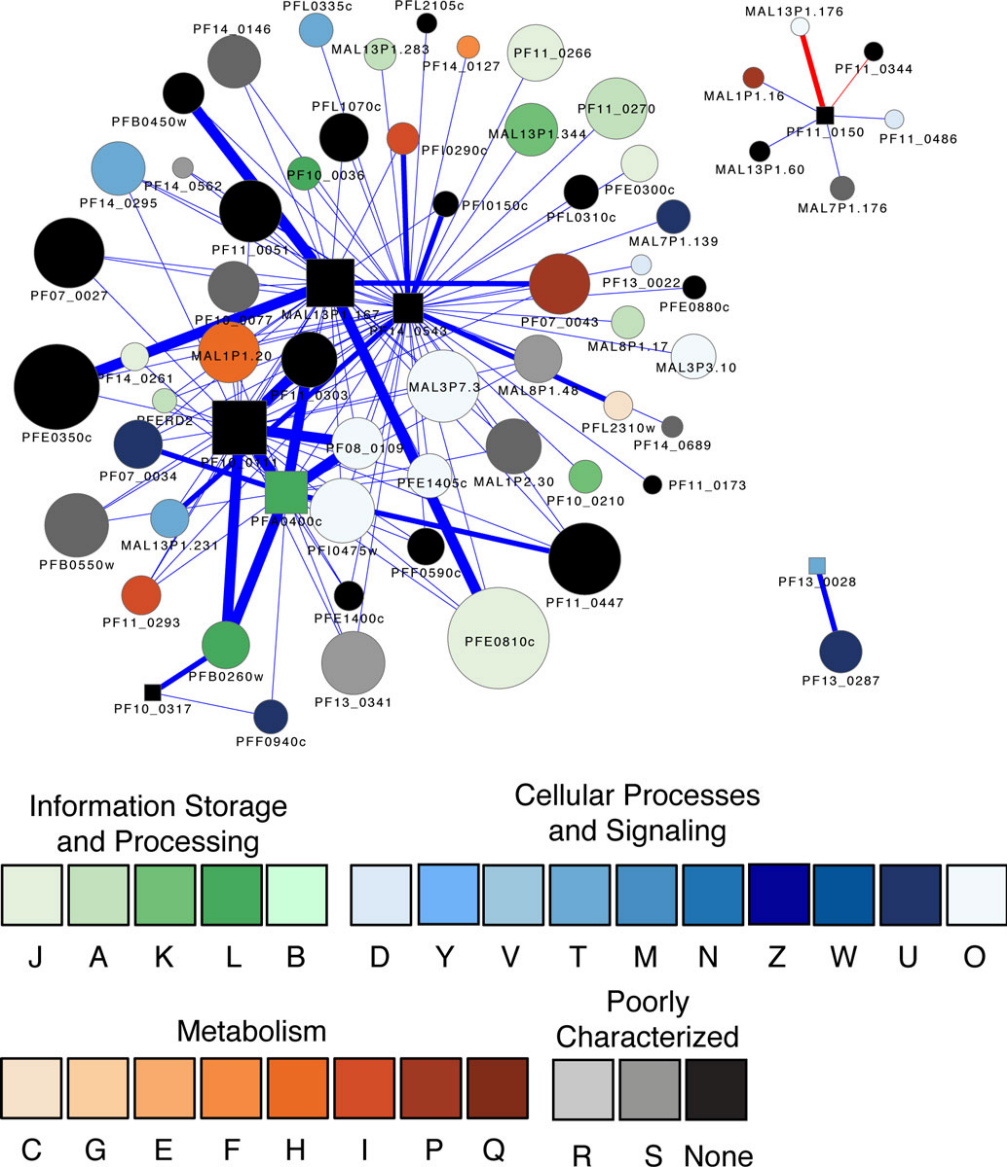
**The set of proteins associated with proteases that carry out regulated intramembrane proteolysis (RIP)**. This novel form of proteolysis is linked to signaling and the associated proteins may be targets for, or mediators of, this system. The red edges indicate experimentally validated interactions; other details of the visualization are as for Figure 2.

The first family, PfAPP (PF14_0543), has 54 association partners (Figure 4 andTable 2). The association partner with the highest confidence score is a putative Rer1 (retrieval receptor for endoplasmic reticulum (ER) membrane proteins, PFI0150c) that is important for localizing proteins to the ER. Another related partner for PfAPP is a putative ER lumen protein retaining receptor (PF13_0280), which contains a signal sequence that facilitates the protein transport between the cis side of the Golgi apparatus and the ER [94]. It is believed that parasite invasion of erythrocytes requires the export of proteins to the ER and the cell surface. They then traverse the parasitophorous vacuole membrane (PVM) into the erythrocyte or parasite-derived membranous structures known as Maurer's clefts. PfAPP, along with the ER-localization proteins, may play a role in protein trafficking, cell-cell communication and remodeling of the host erythrocyte for parasite entry. Other proteins that are associated with PfAPP include secretory proteins, translation initiation and elongation factors, splicing factors and the spliceosome unit, peptide chain release factor, and various enzymes, suggesting it is involved in diverse networks related to transport, translation, posttranslational processing and metabolism. Recent gene disruption assays showed that PfAPP is essential for merozoite invasion and parasite growth [92,93]; the versatile associations of this protease underscore its potential as a drug target.

**Table 2:** Representative *P. falciparum *proteins that are associated with the regulated intramembrane proteolysis (RIP) network.

Protease family	Accession number of protease	Associated Protein accession number	Annotation
A22 (presenilin family)	PF14_0543	PFI0150c	putative retrieval receptor for endoplasmic reticulum membrane proteins
		
		PF13_0280	ER lumen protein retaining receptor
		
		MAL13P1.231	Sec61 alpha subunit, PfSec61
		
		PFB0450w	secretory complex protein 61 gamma subunit
		
		PF11_0447	putative translation initiation factor eIF-1A
		
		PF10_0077	putative eukaryotic translation initiation factor 3 subunit 7
		
		PFL0310c	putative eukaryotic translation initiation factor 3 subunit 8
		
		PFL0335c	putative eukaryotic translation initiation factor 5
		
		PFE1405c	putative eukaryotic translation initiation factor 3, subunit 6
		
		PFC0870w	putative elongation factor 1 (EF-1)
		
		MAL8P1.48	putative splicing factor
		
		PFB0550w	putative peptide chain release factor subunit 1
		
		PF07_0034	cloroquine resistance associated protein Cg3 protein
		
		PF13_0022	cyclin

S54 (Rhomboid family)	PF11_0150	PF11_0344	apical membrane antigen 1
		
		PF11_0486	merozoite adhesive erythrocytic binding protein
		
		PFA0125c	erythrocyte binding antigen-181
		
		MAL13P1.60	erythrocyte binding antigen-140
		
		MAL7P1.176	erythrocyte binding antigen-175
		
		MAL13P1.176	reticulocyte binding protein 2 homolog b

M50 (S2P protease family)	PF13_0028	PF13_0287	adenylosuccinate synthetase
	
	PF10_0317	PFB0260w	putative proteasome 26S regulatory subunit
		
		PFF0940c	putative cell division cycle protein 48 homolog

The second family, PfROM, includes a group of serine proteins with demonstrated roles in parasite invasion [90,91,95,96]. Only one out of the ten rhomboid protease homologs in *P. falciparum*, PfRom1 (PF11_0150), was predicted to have protein-protein associations. Most interestingly, all the six proteins associated with it are antigens that have been considered as vaccine candidates; they belong to three families of adhesins that are essential for parasite invasion (Figure 4 andTable 2): (1) the apical membrane antigen 1 (AMA1, PF11_0344) is an adhesin required for merozoite invasion and it plays an indispensible role in the proliferation and survival of the malaria parasite [97]. PfRom1 was shown to be able to cleave AMA1 [88]; (2) the erythrocyte binding-like (EBL) family is involved in binding to a host chemokine receptor, the Duffy antigen [98]. Among the four EBAs with predicted association with PfRom1, EBA-175(MAL7P1.176) is proven a natural substrate for PfRom1 [88], but it remains unclear whether PfRom1 can cleave EBA-140 (MAL13P1.60), EBA-181 (MAL1P1.16), and a putative merozoite adhesive erythrocytic binding protein (PF11_0486); (3) a reticulocyte binding protein 2 homolog b protein (MAL13P1.176) in the reticulocyte binding-like (RBL) family. PfRom1 is able to cleave the RBL proteins [88]. Apparently, PfRom1 plays a central role in the RIP network that is tightly linked to the invasion process [86] and as such merits further investigation as a drug target.

S2Ps in the third family, PF10_0317 and PF13_0028, have two and one associations, respectively (Figure 4 andTable 2). PF10_0317 is associated with a proteasome 26S regulatory subunit and a cell division cycle (CDC) protein 48 homolog, which is implicated by GO analysis in ER localization and cell cycle regulation. Our previous domain analysis showed that PF10_0317 contains a Der-1 like domain, which was implicated in proteolysis associated with the ER [99-102]. PF13_0028 is associated with an adenylosuccinate synthetase AdsS (PF13_0287), which is important for the *de novo *biosynthesis of purine nucleotides. This association was predicted based on the genome synteny analysis, which revealed that the homologs of S2P and AdsS are located in the same chromosomal neighborhood in a variety of *Actinobacteria*. The functions of these S2Ps in malaria parasites are yet to be defined.

### Parasite egress mediated by proteolysis

**Figure 5:**
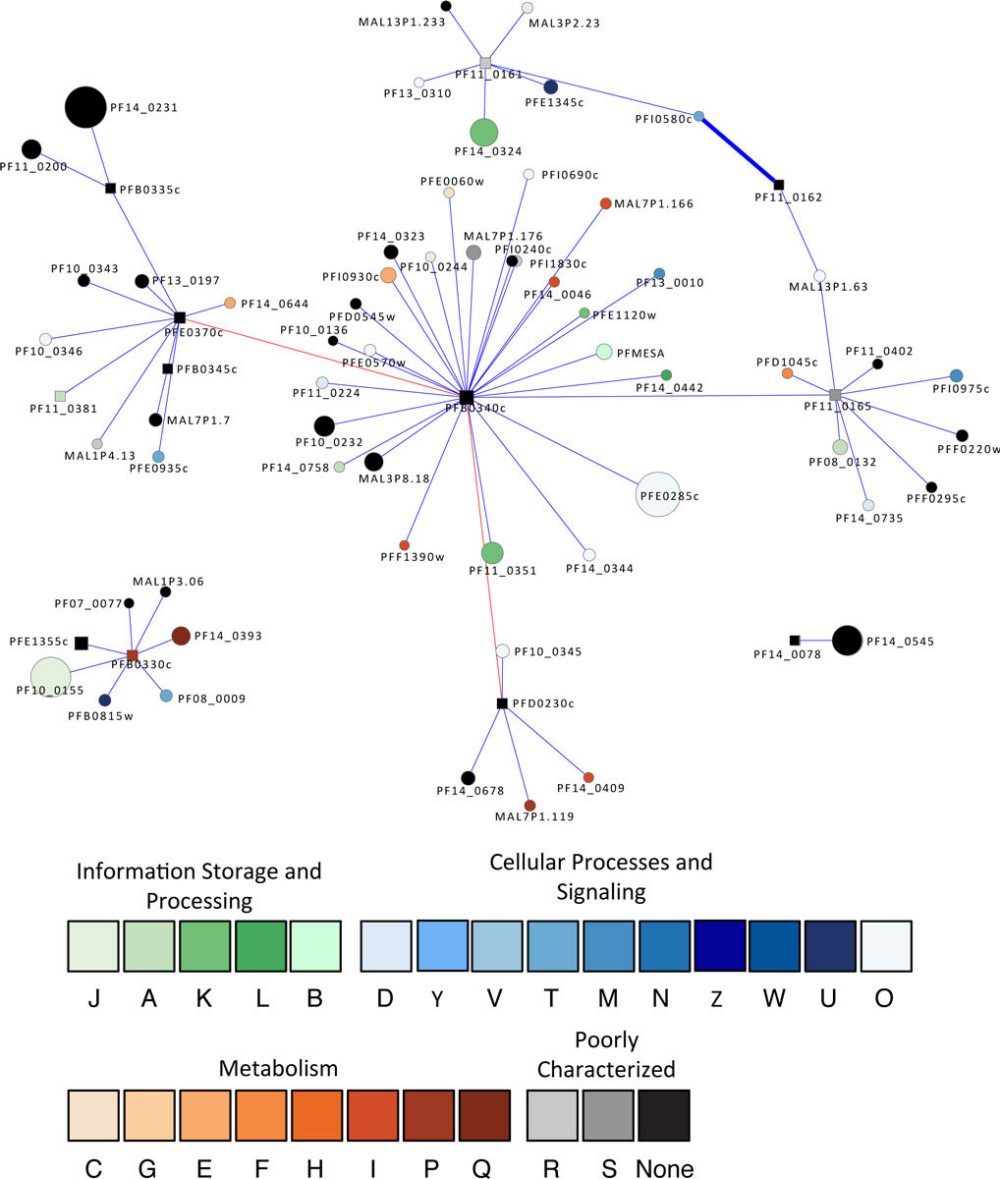
**The protein associations of proteases involved in egress (exit from the erythrocyte)**. SERA5 (PFB0340c) is the most highly connected protease and appears to be a key player. It is linked with two proteases known to activate it, as well as potential substrates (see text). The red edges indicate experimentally validated interactions; other details of the visualization are as for Figure 2.

Egress, the parasite's emergence from host erythrocytes, is a well-coordinated process involving the rupture of the parasitophorous membrane (PVM) and the erythrocyte membrane (EM). Proteases that have been implicated in parasite egress [31,36] include (1) aspartic proteases (plasmepsins PMI, PMII, and PMIII, also known as histo-aspartic protease (HAP)) in the A1 family, (2) cystein proteases in the A1 papain family including falcipain 2a, 2b, and 3, dipeptidyl peptidase 3 (PfDPAP3), and a series of Serine Repeat Antigens (SERAs), and (3) a serine protease subtilase 1 (PfSUB1) in the subtilisin S8 family. We analyzed the protein-association network (Figure 5) involving proteases mediating egress and found that a central player in the network is SERA5 (PFB0340c), which has 28 associations. SERA5 is associated with PfSUB1 (PFE0370c) and PfDPAP3 (PFD0230c). Both these proteases can proteolytically activate SERA5, which triggers downstream processing of cellular substrates [103,104]. SERA5 is also associated with several erythrocyte membrane antigens such as PfEMP2 and EBA-175. It is abundantly expressed in the blood stage, especially in the schizont stage, as revealed by microarray and proteomic analysis. SERA5 has an *in vitro *catalytic activity and it is refractory to gene disruption [105], suggesting its vital role in the parasite life cycle.

### Signal peptidase network

**Figure 6:**
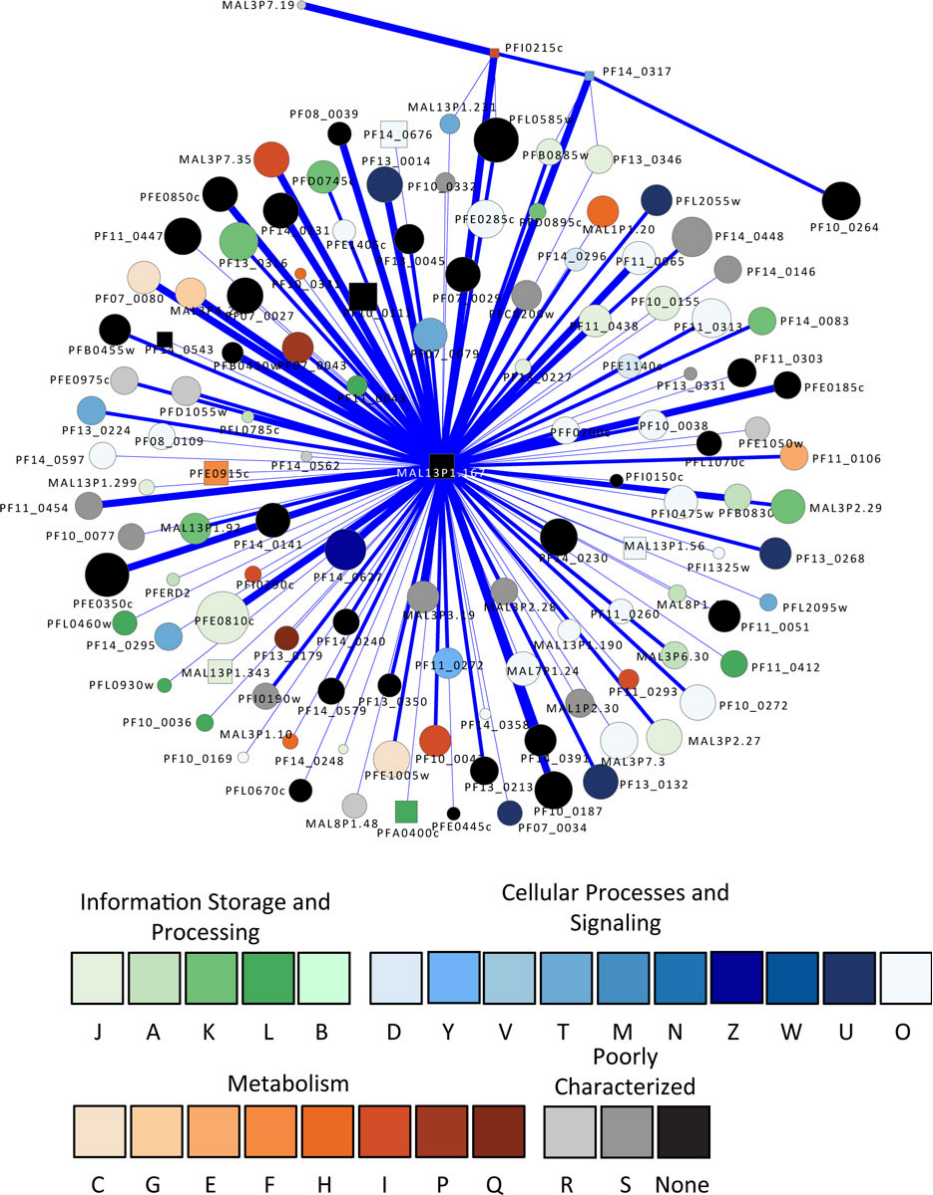
**Proteins associated with three signal peptidases**. As these proteases process signaling sequences on proteins, it is not surprising that they have a wide array of associations. PfSPC21 (MAL13P1.167) is associated with a large number of proteins from COG category O (Posttranslational modification, protein turnover, chaperones). The visualization is as for Figure 2.

As an adaptive survival strategy, the malaria parasite harbors a powerful secretion system that transports parasite-encoded virulence proteins to their subcellular locations. The central players in this secretion system are a group of signal peptidases that are capable of cleaving signal sequences from the target proteins that can then be routed to their destinations. Five signal peptidases have been predicted and characterized, constituting the signal peptidase complex (SPC) in *P. falciparum *[3,106,107]. Three of these peptidases have association partners: PfSPC21 (MAL13P1.167) has 120 associations; the putative microsomal signal peptidase (PF14_0317) has five associations; and the putative SPC22 (PFI0215c) has five associations (Figure 6). The associated proteins are part of the secretion pathway and include secretory complex protein 61 (Pf61) alpha and gamma subunits, a signal recognition particle (SRP) and an SRP receptor, an ER lumen protein retaining receptor, and a transport protein particle (TRAPP) component. These signal peptidases are also associated with members of the ubiquitin-proteasome system and the heat shock response system, with the translational machinery, and with metabolic networks.

### Other potentially important protease-associated networks

Proteases in *P. falciparum *may play other roles important for parasite biology. We previously identified a single copy of calpain PfCalp (MAL13P1.310) in *P. falciparum *genome [3,106,107]. Calpain is crucial for signal transduction, cell cycle regulation, differentiation, development, and cell-cell communication from bacteria to humans. Very little is known about its role in *P. falciparum*. Only four proteins seemed to be associated with calpain: including a putative protein with a C3HC4 type zinc finger, the motif commonly present in transcriptional regulators, a ribosomal protein, and two proteins with unknown function. However, partial knockdown assays recently suggested that PfCalp is essential for the parasite's optimal growth and cell cycle progression [108]. Phylogenetic analysis revealed that PfCalp is a unique type of calpain confined to alveolates (a group of protists) with distant relatedness to human calpains [63,108], adding it to a new line of promising drug target. Another class of proteases that mediate cell cycle regulation and programmed cell death is comprised of the three metacaspases from the C14 protease family [63,109]. Only one association partner was identified for PF13_0289 and PF14_0363, (polyubiquitin and a hypothetical protein with unknown function respectively), and no associations were found for PF14_0160, reflecting our limited knowledge about their functions in malaria parasite.

## Conclusions

Our network analysis of proteases from *P. falciparum *uses a so-called guilt-by-association approach to extract sets of proteins from the proteome that are candidates for further study. The network biology approach is readily adapted to any system for which a genome sequence exists and for which some type of protein-protein association is available, although there are limitations. Some of these stem from missing data, and/or noisy data, which lead to underestimation of the S value for a pair of associated proteins, but this problem becomes less significant with each release of data. A second problem is the lack of any dynamic element in evaluating the associations. A more formal integration of expression data could help to ameliorate this situation, especially expression data sets gathered under different conditions. Despite these limitations, our results produced known associations, which serve as positive controls such as the ubiquitin-proteasome system (UPS). It also indicated that proteases are playing previously unrecognized role in the biology of the parasite, such as the proteases that mediate the stress responses. Our results also imply that certain of these proteases, such as the proteases that mediate regulated intramembrane proteolysis, parasite egress, and signal peptide processing and protein secretion, may be good candidates for antimalarial targeting, as they are highly connected in the network. Furthermore, some of these candidates are known to have no or only distantly related homologs in humans, which reduces the probability of adverse effects resulting from their inactivation. Finally, our analysis has identified new components of previously recognized systems in the parasite, such as the protein(s) involved in transcriptional regulation, cell cycle progression, invasion, protein trafficking, and immune evasion in the UPS, or the antioxidant defense proteins associated with the heat shock response systems.

## Methods

### The protease data

The proteases in *P. falciparum *were predicted using a comparative genomics approach and a support vector machine (SVM)-based, supervised machine learning approach [1-3]. The classification and annotation were according to the MEROPS protease nomenclature, which is based on intrinsic evolutionary and structural relationships [110].

### Network data and analysis

The complete set of protein-protein associations for *P. falciparum *was extracted from the downloaded STRING database [4]; each association between a pair of proteins has a confidence score (S) ranging from 0.15 to 0.999 that was inferred from the evidence used to establish the association, such as homology transfer, KEGG pathway assignments, conserved chromosome synteny, phylogenetic co-occurence, and literature co-occurence [111]. This set of associations was visualized in Cytoscape [112] and converted to an undirected weighted graph, where there is a single edge between any pair of proteins and the S value is used as the weight. The network was characterized using NetworkAnalyzer [113] and significant modules were detected using MINE [114] and MCODE [115]. The default values were used for all three plugins. The set of proteins directly associated with the 77 proteases in the association set were screened using BiNGO [116] to determine if any categories of proteins, as identified by their Gene Ontology terms, were over-represented. The hypergeometric test was used with the Benjamini and Hochberg false discovery date correction. A significance level of 0.05 was selected.

### The omics data mining

We downloaded the *P. falciparum *genomic sequence and annotation data [18], transcriptomic microarray data [6-8], mass-spectrometry proteomic data [9-12], and protein-protein interactome [5] data for network associated proteins from PlasmoDB, the Plasmodium Genome resource center (http://www.plasmodb.org) [117]. Conserved domains/motifs in *P. falciparum *sequences were identified by searching InterPro [118]. Multiple alignments were obtained using the ClustalX program [119] and T-coffee [120], followed by manual inspection and editing. Phylogenetic trees were inferred by the neighbor-joining, maximum-parsimony and maximum-likelihood methods, using MEGA5 [121].

## List of abbreviations used

AMA: apical membrane antigen; CDPK: calcium-dependent protein kinase; DOXP: 1-deoxy-D-xylulose 5-phosphate; EBL: erythrocyte binding-like; EF: elongation factor; EM: erythrocyte membrane; ER: endoplasmic reticulum; GO: Gene Ontology; HAP: histo-aspartic protease; HSP: heat shock protein; MBF: multiprotein bridging factor; ORF: open reading frame; PPI: protein-protein interaction; PVM: parasitophorous vacuole membrane; RBL: reticulocyte binding-like; RIP: regulated intramembrane proteolysis; SERA: Serine Repeat Antigen; SKP: S-phase kinase-associated protein; SOD: superoxide dismutase; SPAM: secreted polymorphic antigen associated with merozoites; SPC: signal peptidase complex; SVM: support vector machine; TBP: TATA box-binding protein; TMH: transmembrane-spanning helical; UPS: ubiquitin-proteasome system; Y2H: yeast 2-hybrid

## Competing interests

The authors declare that they have no competing interests.

## Authors' contributions

TGL and YW conceived and designed the study, performed bioinformatics data analysis, and drafted the manuscript. HC wrote scripts, and ZZ helped with data analysis. All authors read and approved the final manuscript.

## Supplementary Material

Additional file 1 ***P. falciparum *proteases and their degrees of connectivity in protein association networks**. Click here for file

Additional file 2 **The protein-protein associations involving proteases in *Plasmodium falciparum***. Click here for file

Additional file 3 **Functional categories involving protease associations identified by Gene Ontology enrichment analysis**. Click here for file

Additional file 4 **The graph shows the set of proteins associated with the proteases thought to be part of the *P. falciparum *ubiquitin-proteasome protein degradation system**. Nodes are colored according to their functional classification in the eggNOG database [122] (key is shown). Node size is proportional to the degree of the node. Confidence scores for the interactions among the nodes (S values from STRING) were divided into three groups - low (0.150-0.399), medium (0.400-0.700) and high (0.701-0.999); the groups are represented by thin, medium and heavy lines, respectively. Click here for file
