# Supplementary material for: Protease-associated cellular networks in malaria parasite Plasmodium falciparum
Source: BMC Genomics. 2011 Dec 23;12(Suppl 5):S9. doi: 10.1186/1471-2164-12-S5-S9 (PMC3287505; doi:10.1186/1471-2164-12-S5-S9)
Supplement: Additional file 1 — P. falciparum proteases and their degrees of connectivity in protein association networks. [file 1471-2164-12-S5-S9-S1.docx]

**Additional file 1. *P. falciparum* proteases and their degrees of connectivity in protein association networks.**

| **Catalytic Type** | **Protease Family** | ***P. falciparum*** | **Degree of Connectivity** |
| --- | --- | --- | --- |
| **Aspartic** | A1 (pepsin family) | PF14_0078 | 1 |
|  |  | PF08_0108 | 3 |
|  | A22 (presenilin family) | PF14_0543 | 53 |
| **Cysteine** | C1 (papain family) | PF11_0165 | 9 |
|  |  | PF11_0162 | 2 |
|  |  | PF11_0161 | 6 |
|  |  | PFD0230c | 5 |
|  |  | PF11_0174 | 2 |
|  |  | PFB0330c | 7 |
|  |  | PFB0335c | 1 |
|  |  | PFB0340c | 28 |
|  |  | PFB0345c | 2 |
|  | C2 (calpain family) | MAL13P1.310 | 4 |
|  | C12 (ubiquitin C-terminal hydrolase family) | PF14_0576 | 6 |
|  |  | PF11_0177 | 11 |
|  | C13 (legumain family) | PF11_0298 | 6 |
|  | C14 (caspase family) | PF13_0289 | 1 |
|  |  | PF14_0363 | 1 |
|  | C19 (ubiquitin-specific protease family) | PFE1355c | 22 |
|  |  | PFE0835w | 3 |
|  |  | MAL7P1.147 | 9 |
|  |  | PFI0225w | 25 |
|  |  | PF13_0096 | 3 |
|  |  | PFD0655c | 3 |
|  | C48 (Ulp1endopeptidase family) | PFL1635w | 1 |
|  | C50 (separase family) | MAL8P1.113 | 2 |
| **Metallo** | M1 (aminopeptidase N family) | MAL13P1.56 | 100 |
|  | M3 (thimet oligopeptidase) | PF10_0058 | 2 |
|  |  | MAL13P1.184 | 1 |
|  | M16 (pitrilysin family) | PFE1155c | 10 |
|  |  | PFI1625c | 78 |
|  |  | PF11_0189 | 1 |
|  |  | PF13_0322 | 5 |
|  | M17 (leucyl aminopeptidase family) | PF14_0439 | 73 |
|  | M18 (aminopeptidase I) | PFI1570c | 17 |
|  | M22 (O-sialoglycoprotein peptidase) | PF10_0299 | 17 |
|  |  | PFD0440w | 1 |
|  | M24 (methionyl aminopeptidase 1) | PFE1360c | 75 |
|  |  | PF10_0150 | 31 |
|  |  | PF14_0327 | 70 |
|  |  | PF14_0517 | 28 |
|  | M41 (FtsH endopeptidase family) | PF11_0203 | 13 |
|  |  | PFL1925w | 30 |
|  |  | PF14_0616 | 54 |
|  | M50 (S2P protease family) | PF13_0028 | 1 |
|  |  | PF10_0317 | 2 |
|  | M67 (Poh1 peptidase) | MAL13P1.343 | 114 |
|  |  | PFI0895c | 32 |
|  |  | PFI0630w | 60 |
| **Serine** | S8 (subtilisin family) | PFE0370c | 10 |
|  |  | PF11_0381 | 3 |
|  |  | PFE0355c | 1 |
|  | S14 (ClpP endopeptidase family) | PFC0310c | 69 |
|  |  | PF14_0063 | 2 |
|  |  | PF14_0348 | 54 |
|  |  | PF08_0063 | 65 |
|  |  | PF11_0175 | 70 |
|  | S16 (lon protease family) | PF14_0147 | 24 |
|  | S26 (signal peptidase I family) | MAL13P1.167 | 120 |
|  |  | PF14_0317 | 5 |
|  | S54 (Rhomboid family) | PF11_0150 | 6 |
| **Threonine** | T1 (proteasome family) | PF14_0716 | 54 |
|  |  | PFF0420c | 70 |
|  |  | PF13_0282 | 65 |
|  |  | PF07_0112 | 59 |
|  |  | MAL8P1.128 | 54 |
|  |  | MAL13P1.270 | 58 |
|  |  | PFE0915c | 114 |
|  |  | MAL8P1.142 | 115 |
|  |  | PFA0400c | 99 |
|  |  | PF14_0676 | 132 |
|  |  | PFI1545c | 48 |
|  |  | PF13_0156 | 55 |
|  |  | PF10_0111 | 143 |
|  |  | PFL1465c | 80 |
| **Unknown** | Zinc protease | PF13_0260 | 3 |
|  | Signal peptidase | PFI0215c | 5 |
