# Supplementary material for: Protease-associated cellular networks in malaria parasite Plasmodium falciparum
Source: BMC Genomics. 2011 Dec 23;12(Suppl 5):S9. doi: 10.1186/1471-2164-12-S5-S9 (PMC3287505; doi:10.1186/1471-2164-12-S5-S9)
Supplement: Additional file 4 — The graph shows the set of proteins associated with the proteases thought to be part of the P. falciparum ubiquitin-proteasome protein degradation system. Nodes are colored according to their functional classification in the eggNOG database [122] (key is shown). Node size is proportional to the degree of the node. Confidence scores for the interactions among the nodes (S values from STRING) were divided into three groups - low (0.150-0.399), medium (0.400-0.700) and high (0.701-0.999); the groups are represented by thin, medium and heavy lines, respectively. [file 1471-2164-12-S5-S9-S4.pdf]

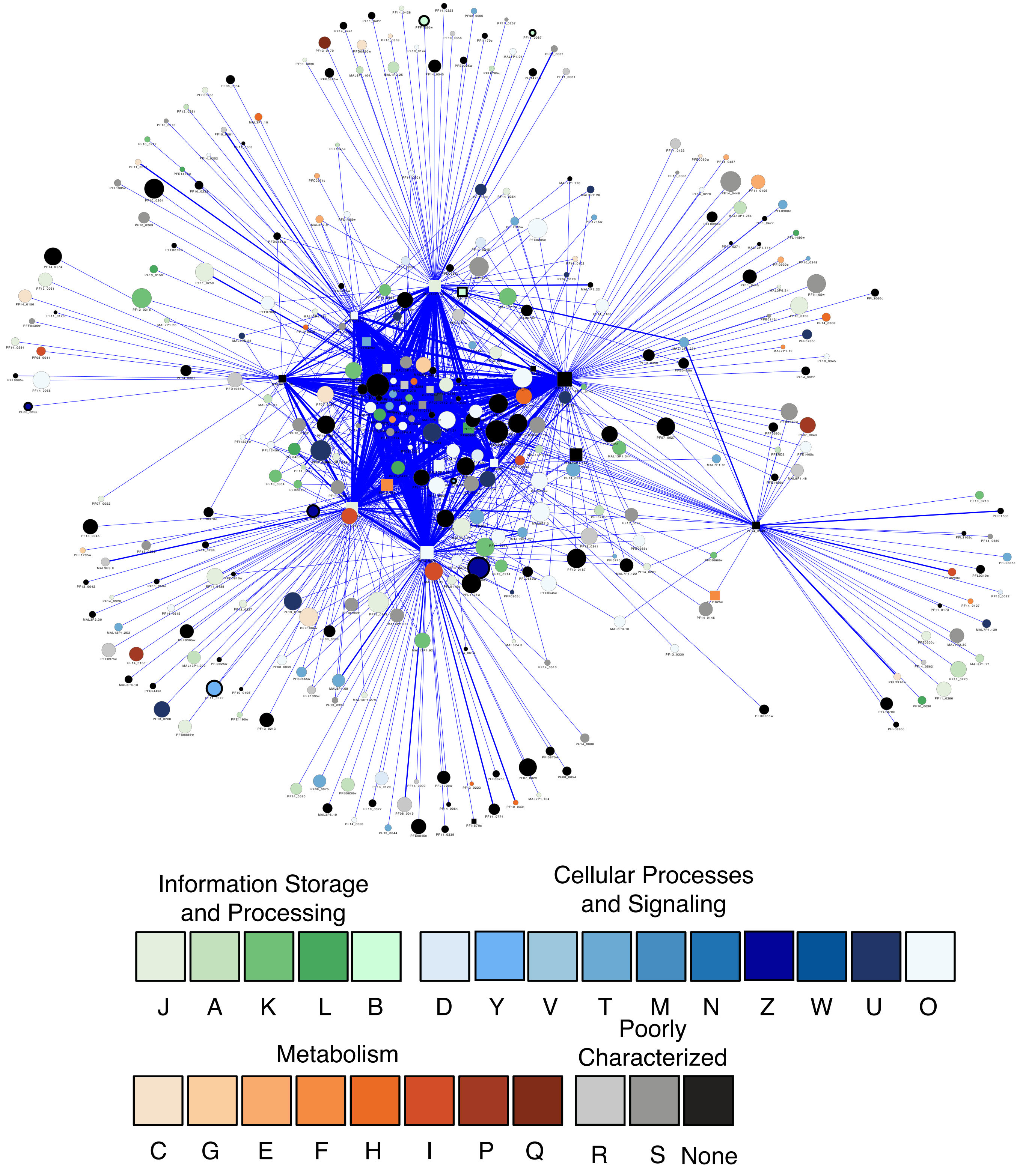
Information Storage and Processing
J
A
K
L
B
Cellular Processes and Signaling
D
Y
V
T
M
N
Z
W
U
O
Metabolism
C
G
E
F
H
I
P
Q
Poorly Characterized
R
S
None
